# Regulatory Role of Phospholipids in Hepatitis C Virus Replication and Protein Function

**DOI:** 10.3390/pathogens11010102

**Published:** 2022-01-15

**Authors:** Anna V. Bulankina, Rebecca M. Richter, Christoph Welsch

**Affiliations:** 1 Department of Internal Medicine 1, Goethe University Hospital Frankfurt, 60590 Frankfurt, Germany; Anna.Bulankina@kgu.de (A.V.B.); Rebecca.Richter@kgu.de (R.M.R.); 2 Research Group “Molecular Evolution & Adaptation”, 60590 Frankfurt, Germany

**Keywords:** RNA virus, hepatitis C virus, phospholipid, viral replication, membrane remodeling, interactions, NS5A

## Abstract

Positive-strand RNA viruses such as hepatitis C virus (HCV) hijack key factors of lipid metabolism of infected cells and extensively modify intracellular membranes to support the viral lifecycle. While lipid metabolism plays key roles in viral particle assembly and maturation, viral RNA synthesis is closely linked to the remodeling of intracellular membranes. The formation of viral replication factories requires a number of interactions between virus proteins and host factors including lipids. The structure–function relationship of those proteins is influenced by their lipid environments and lipids that selectively modulate protein function. Here, we review our current understanding on the roles of phospholipids in HCV replication and of lipid–protein interactions in the structure–function relationship of the NS5A protein. NS5A is a key factor in membrane remodeling in HCV-infected cells and is known to recruit phosphatidylinositol 4-kinase III alpha to generate phosphatidylinositol 4-phosphate at the sites of replication. The dynamic interplay between lipids and viral proteins within intracellular membranes is likely key towards understanding basic mechanisms in the pathobiology of virus diseases, the mode of action of specific antiviral agents and related drug resistance mechanisms.

## 1. Introduction

**Figure 1:**
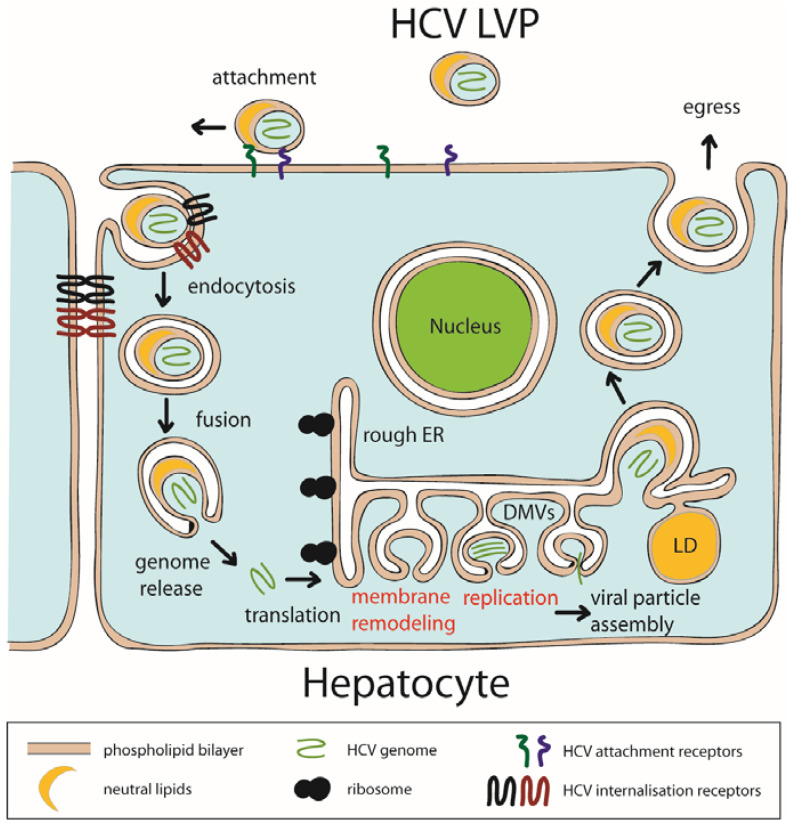
Schematic representation of the HCV life cycle. Steps of the HCV genome replication cycle involving interactions between nonstructural viral proteins and host phospholipids are shown in red. Interaction of HCV lipo-viro-particles (LVPs) with hepatocytes is mediated by attachment receptors, followed by tight binding to internalization receptors, which are required for HCV endocytosis. After fusion of the enveloping HCV-LVP membrane with the endosome, the HCV +RNA genome is released into the cytoplasm and translated at the ER. HCV proteins interact with the membrane of the ER and induce membrane remodeling and formation of replication organelles (comprising double-membrane vesicles, DMVs). Viral assembly occurs at detergent-resistant membranes (DRMs) of the ER in close proximity to DMVs and cytoplasmic lipid droplets (LDs). The maturing LVPs are finally released via the secretory pathway.

Positive-sense single-stranded RNA (+RNA) viruses include important human pathogens such as the hepatitis C virus (HCV) and coronaviruses. HCV belongs to the *Flaviviridae* family, that also comprises Dengue, Zika, West Nile, Yellow Fever and Japanese Encephalitis viruses. Members of this heterogeneous group of viruses modify host cell membranes and lipid metabolism in many aspects to support viral RNA replication [1,2]. Like in all +RNA viruses, the HCV genome is synthesized by a multi-protein replicase complex that assembles in association with intracellular membranes. Known as the ‘membranous web’ (MW) in HCV-infected cells [3,4], this specialized compartment provides a platform for viral RNA synthesis. Its membranes are derived from ER, but become enriched in cholesterol, sphingolipids and phosphatidylinositol 4-phosphate (PI4P). The MW contains mainly double membrane vesicles (DMVs) and multimembrane vesicles (MMVs). The induction of DMVs, serving as replication organelles (Figure 1) [5], is paralleled by reprogramming the lipid metabolism of infected cells [6,7]. These alterations require function of HCV proteins, which remodel host cell membranes to form replication organelles and recruit enzymes of the lipid metabolism. Both these processes require a concerted interaction of virus and host proteins as well as lipids. However, functional studies of viral proteins in their natural lipid environment are particularly difficult and, despite the key role of lipids in the HCV life cycle and pathogenesis, dynamic models to address the role of the lipid environment for structural rearrangements in HCV proteins are still in their infancy.

In this review we will summarize our current understanding of the role of phospholipids, in particular negatively charged phospholipids (NCPs), in formation of the HCV-induced MW and the replication of HCV RNA.

## 2. HCV Genome Replication

### 2.1. HCV Structural and Nonstructural Proteins

The HCV RNA genome contains three major parts: a single open reading frame (ORF) flanked by 5’ and 3’ untranslated regions (UTRs). The ORF encodes a viral polyprotein, which comprises a total of ten structural and nonstructural (NS) proteins cleaved by viral and host proteases. The ORF can be divided into two modules according to the roles of the encoded proteins in the viral life cycle: 5’- or ‘assembly’ module (core, E1, E2, p7 and NS2) and 3’- or ‘replication’ module (NS3, NS4A, NS4B, NS5A and NS5B) (reviewed in [8,9]). The minimal part of HCV genome sufficient for translation and replication includes both UTRs and the ‘replication’ module [10]. The 5’-UTR comprises an internal ribosomal entry site (IRES) (described in detail in [11]) that is essential for translation. All proteins of the replication module participate in viral particle assembly.

Structural proteins of the ‘assembly’ module include core protein, building up the viral capsid, and envelope glycoproteins E1 and E2, inserted into the surrounding capsid membrane. The NS protein p7 is a viroporin that forms an intracellular proton-conducting transmembrane channel, supporting virus assembly and release by shunting the pH of intracellular compartments. NS2 flanks the ‘assembly’ module on the 3’-end and is responsible for the polyprotein cleavage on the NS2 and NS3 junction and serves as a hub for the infectious viral particles assembly [9]. 

Viral nonstructural proteins are important targets for specific antiviral drugs. Lipid-exposed surfaces of these proteins play an important, albeit poorly understood, role in the development of drug resistance. Under drug pressure, viruses can develop escape mechanisms through mutations (resistance mutations). In order to nevertheless ensure correct structure and function of the proteins thus altered, so-called second-site mutations are observed to occur within protein–lipid interaction domains, among others. Such exchanges can, for example, compensate for deficits in the replicative fitness of drug-resistant viral variants. The NS3–4A protease/helicase plays an essential role in the HCV replication cycle and is a prime target site for direct antiviral therapy [8]. Proteolytic processing of the viral polyprotein by NS3-4A protease is a prerequisite for the viral RNA replication. This is why inhibitors against the NS3-4A protease have proven to be useful drugs in the treatment of HCV infection. Although a large number of drug-resistant viral variants with exchanges within the protease have been detected, very few compensatory second-site exchanges have been identified that compensate for fitness deficits that occur in the course of drug resistance [12,13,14,15]. NS4B is a highly hydrophobic protein which is capable of inducing intracellular membrane rearrangements (formation of single membrane vesicles) [16,17]. NS5A is a multifunctional phospho- and metalloprotein without known enzymatic activity. NS5A is capable of triggering DMVs formation and, possibly, acting as a master regulator of viral RNA translation, replication and assembly of infectious viral particles [5,9,18]. A study on NS5A dimer interface residues and their role in protein conformational rearrangements, NS5A oligomerization and interaction with host factors finds NS5A self-interactions critical for HCV RNA replication and infectious virus production. In turn, NS5A self-interactions were shown to be dependent on NS5A hyperphosphorylation, subcellular localization, as well as interaction with host proteins such as Cyclophilin A. The data provided evidence that surface exposed NS5A residues engaged in two different dimeric interactions in crystal structures are involved in NS5A self-interactions in cells and supported functional significance of NS5A oligomers formed via multiple interfaces during the HCV replication cycle [19]. NS5A inhibitors are central to most direct-acting antiviral agent (DAA)-treatment approaches and retreatment options against HCV [20,21,22,23]. Despite the absence of enzymatic activity but due to its key role in establishment of the HCV replication compartment, significance for viral particle assembly, and tendency to oligo- or polymerize, NS5A turned out to be an excellent therapeutic target: NS5A inhibitors can have exceptional potency (in pM range) (see below) [24,25,26]. NS5B is an RNA-dependent RNA polymerase, which have been also successfully targeted by DAAs [8,10,25]. The accumulated knowledge about nucleotide analogs and NS5B inhibitors contributed to the development of a drug against severe acute respiratory syndrome coronavirus 2 (SARS-CoV-2) [27]. 

### 2.2. HCV Replication Organelle

Formation of the replication organelles is a prerequisite for efficient viral RNA replication [28]. It is believed that DMVs are the HCV replication organelles because (i) they contain nonstructural HCV proteins required for RNA replication [3,4,29,30]; (ii) there are indications from electron microscopy experiments with immunogold labelling that double-stranded RNA is present within DMVs [29]; (iii) HCV replication complexes are resistant to protease and nuclease activity [31,32,33]; and iv) DMV formation in infected cells peaks simultaneously with viral RNA replication [17]. Accordingly, functions of membranous organelles supporting viral replication may include (i) shielding viral single-stranded RNAs and double-stranded RNA replication intermediates from innate immunity (reviewed in [34]) and (ii) creating a unique environment for concentration of viral and host factors required for viral RNA replication and its spatiotemporal control [35]. The replication organelles are continuously generated *de novo* at spatially distinct sites from existing ones [36,37]. It is shown that NS5A is sufficient to induce DMVs in cultured cells, however, the number of DMVs increases dramatically when all proteins of the viral ‘replication’ module are co-expressed [5]. Coordinated interactions of viral and host factors are required and closely regulated for formation of the MW with specialized lipid composition comprising the enrichment in PI4P [38,39], cholesterol and sphingolipids [4,7,17,31] as well as membranous phospholipids with longer and more desaturated fatty acids that play key roles in membrane curvature and fluidity [7,40].

### 2.3. Role of Phospholipids in HCV Genome Replication

#### 2.3.1. Direct Interaction of NS Proteins with Phospholipids and Stimulation of Replication

All proteins of the ‘replication’ module of HCV are directly or indirectly associated with intracellular membranes and hence specifically interact with lipids or bear a potential for specific interaction with phospholipids. The NS4B protein selectively binds to NCPs that are phosphatidylinositol phosphates (PI4P, phosphatidylinositol 4,5-bisphosphate (PIP2) and phosphatidylinositol 3,4,5-trisphosphate (PIP3)), phosphatidylglycerol, phosphatidylserine and cardiolipin (Table 1). NS4B interacts via its N-terminal amphipathic helix (AH) 2 with charged headgroups of phospholipids [41,42]. These interactions alter the membrane morphology and promote dissociation of AH2 oligomers [42]. However, only little is known about the role of these interactions in HCV replication. 

**Figure 2:**
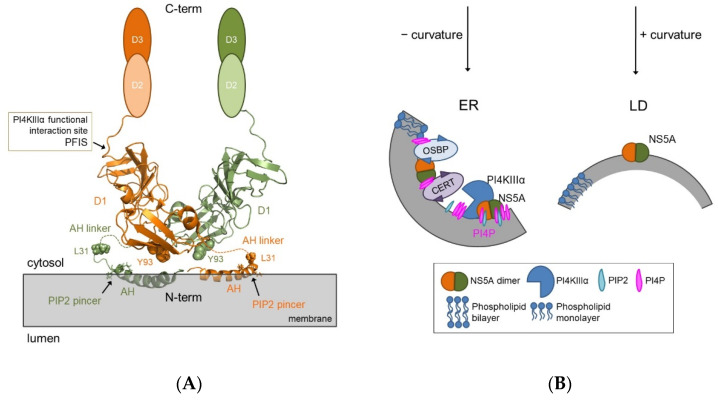
Hypothetical models of the NS5A interaction with membranes. (**A**) NS5A model of a full-length dimer comprising N-terminal membrane attachment portions—amphipathic α-helices (AHs) based on Protein Data Bank (PDB) entries 1ZH1 for NS5A D1 dimer and 1R7G for AH. Domains (D) 2 and 3 of NS5A are intrinsically unfolded and likely interact with multiple host factors. The N-terminal AH specifically interacts with phosphatidylinositol 4,5-bisphosphate (PIP2) via a pincer motif (residues as stick models). Domain 1 of NS5A interacts with the lipid kinase phosphatidylinositol 4-kinase III alpha (PI4KIIIα) via PFIS motif; (**B**) Binding of NS5A at the replication complex (negative membrane curvature) or lipid droplets (LD) (positive membrane curvature). NS5A-mediated subversion of lipid metabolism at the replication complex by sequential recruitment and activation of PI4KIIIα and oxysterol binding protein (OSBP) with accumulation of PI4P and subsequent exchange for cholesterol by OSBP or for ceramide by ceramide transfer protein (CERT).

Similarly, the N-terminal AH of NS5A directly and specifically interacts with the NCP PIP2 [40,41]. The PIP2 interaction is mediated by a basic amino acid PIP2 pincer (BAAPP) motif comprising two positively charged residues, Lys20 and Lys26 (Figure 2A). The BAAPP motif in NS5A is conserved over all HCV genotypes and is also found in NS4B AH1, some host proteins (e.g., apolipoprotein B) and proteins of other pathogens, such as the core protein of the Japanese Encephalitis virus. The binding of NS5A AH to PIP2 results in conformational changes of the helical structure of NS5A AH with consecutive stabilization of an interaction to the host factor TBC1D20, a guanosine triphosphatase activating protein for Rab1. The association between NS5A and PIP2 takes place in cells replicating HCV RNA only and is crucial for the latter process [43]. Interestingly, conserved positively charged residues flanking NS4B AH1 play an important role in HCV genome replication by controlling the size of DMVs [46], however, to date direct interaction of NS4B AH1 with NCPs has not been demonstrated.

NS5B specifically binds sphingomyelin (SM), which increases NS5B RNA polymerase activity most efficiently in genotype 1b HCV [45,47,48]. NS5B harbors an SM binding domain (SBD, Gly230-Gly263), which is a helix-turn-helix structure motif very similar among HCV genotypes [45]. It is likely that NS5B undergoes structural rearrangements upon SM binding to SBD to promote RNA binding. Based on reaction kinetics, it was suggested that 20 SM molecules are needed to interact with SBD of NS5B for activation of its polymerase activity [47]. Docking experiments showed interaction of SM molecules with SBD forming a salt bridge with residue 244 and hydrogen bonds with residues 241, 242 and 250. Based on the modeling, binding of a single SM molecule per SBD is supported [49].

#### 2.3.2. Indirect Effects of Phospholipids on Replication Complex Assembly

The most studied indirect role of phospholipids and in particular NCPs (phosphatidylinositol 3-phosphate (PI3P) and PI4P) on HCV RNA replication is the recruitment of host effector proteins (see below) to nascent replication organelles for further modification of their composition and membrane remodeling for DMV formation. Cellular membranes are laterally heterogeneous with distinct biophysical properties and lipid compositions. HCV replication is associated to detergent-resistant membranes (DRMs) similar to lipid rafts [4,31,50]. The lipid raft is a subtype of lateral membrane heterogeneity enriched in specific lipids, in particular cholesterol, saturated phospholipids, SM and glycosylated lipids, that drive the formation of ordered membrane regions that recruit other, specialized lipids and proteins [51]. DRM destabilization or disruption results in the loss of integrity of the HCV replication complex and disrupts interactions of viral NS proteins [45,52]. So far, accumulation of PI4P in replication organelles is predominantly considered as an intermediate step in viral replication and a prerequisite for accumulation of cholesterol and SM in HCV replication organelles (Figure 2B). To this end, NS5A sequentially recruits and activates host phosphatidylinositol 4-kinase III alpha (PI4KIIIα) and oxysterol binding protein (OSBP) to the MW [38,53]. The former synthesizes PI4P from phosphatidylinositol that results in accumulation of PI4P in MWs, which is exchanged for cholesterol by OSBP or for ceramide (SM synthesis precursor) by ceramide transfer protein (CERT) [52,53] (Figure 2B). Both OSBP and CERT are nonvesicular lipid transporters which exchange PI4P and cholesterol or ceramide, respectively, at membrane contact sites [54].

Besides direct binding and activation of RNA-dependent RNA polymerase NS5B, as discussed above, SM also plays a structural role in formation of DMVs. It was shown that DMV formation is impaired by suppression of SM biosynthesis with small-molecule inhibitors in HCV replicon cells or by establishment of a CERT knockout cell line followed by transfection with HCV replicon or constructs not supporting HCV replication [52]. An accumulation of some glycosphingolipids triggered by HCV infection coincided with relocalization to the MW of another host PI4P-effector, four-phosphate adapter protein 2 (FAPP2). Mutations in the glycosphingolipid-binding domain of FAPP2 inhibit HCV RNA replication, while siRNA depletion of FAPP2 leads to the loss of punctate NS5A and NS4B staining and, likely, disruption of HCV replication complexes [55].

Chemical inhibition of class III phosphatidylinositol 3-kinase (PI3K) complex or knock down of its catalytic subunit impedes HCV replication and DMVs formation, likely via suppression of activity of PI3P effector double FYVE-containing protein 1 (DFCP1) [56,57]. DFCP1 induces formation of PI3P-dependent cup-shaped protrusions of ER membrane during early stage autophagocytosis and potentially promotes membrane remodeling required for HCV genome replication [57,58].

An intimate connection between the biology of phospholipids and the function of viral proteins is also demonstrated by oxidative stress during HCV infection caused by both host inflammatory responses and direct interactions of viral proteins with mitochondria. Lipid peroxides are formed on polyunsaturated fatty acid chains of phospholipids within membranes by reactive intermediates produced during oxidative stress. They alter membrane fluidity and permeability and potentially contribute to a variety of disease states [59]. The degradation products of these lipid peroxides include reactive aldehydes, such as acrolein, 4-hydroxy-2-nonenal and malondialdehyde, that add to this damage by forming adducts with membrane proteins, thereby modulating their biological activities [59,60]. It is noteworthy that the HCV replicase is uniquely regulated by lipid peroxidation. HCV possesses a unique capacity to sense lipid peroxides induced by infection, and to respond to their presence by restricting viral RNA synthesis, thereby limiting virus replication and possibly facilitating virus persistence. Yamane et al. showed that endogenous oxidative membrane damage lowers the 50% effective concentration of DAAs in vitro, suggesting critical regulation of the conformation of the membrane-bound portions of the NS3-4A protease and the NS5B polymerase [61].

## 3. Reprogramming/Hijacking Host PI4KIIIα

The morphological reorganization of cellular membranes and changes in the lipidome of HCV infected cells result from hijacking and reprogramming of the host cell lipid metabolism by the virus. These alterations are induced by activation of sterol regulatory element-binding protein (SREBP) pathway [62] and direct interaction of HCV proteins with several host proteins such as the fatty acid synthase [63,64] and phosphatidylinositol 4-kinases [38,39,65,66,67,68]. The latter deserve special attention. After HCV infection, a more than 3-fold increase in total cellular concentration of PI4P compared to uninfected cells was observed [38,39,68]. It is noteworthy that PI4P at physiologically relevant concentrations (2 mol%) bears a potential to induce membrane curvature [69].

In uninfected cells, PI4P is predominantly found in Golgi membranes and in the inner leaflet of the plasma membrane, while a smaller PI4P population exists on ER exit sites [70,71]. Both latter pools of PI4P are produced by PI4KIIIα, which is primarily recruited to these compartments [72,73]. The subcellular localization of PI4KIIIα is altered upon HCV infection by interactions with NS5A and NS5B and recruitment to the MW, thereby increasing the PI4P levels in the replication organelles and decreasing them on the plasma membrane [38,67,73,74]. The kinase activity of PI4KIIIα is enhanced through the interaction with NS5A [38,39,67], and even ectopic expression of NS5A alone increases PI4P levels within the cell by PI4KIIIα activity [75]. The PI4KIIIα functional interaction site (PFIS) in NS5A is located at the very end of domain 1 (amino acids (aa) 202-210) [76] (Figure 2A), which is not resolved in the published crystal structures [77,78,79]. Mutations in PFIS lead to hyperphosphorylation of NS5A [76]. It is likely that PI4KIIIα possesses multiple, possibly redundant NS5A-binding sites [80]. NS5A–PI4KIIIα interaction may depend on human choline kinase-α (hCKα) [81]. Treatment with NS5A inhibitors (NS5Ai) leads to an impairment in the NS5A-PI4KIIIα complex formation that is paralleled by a significant reduction in PI4P and cholesterol levels within the MW [82]. A similar decrease is also observed upon treatment with PI4KIIIα-targeting inhibitors [38,73]. Knockdown of PI4KIIIα significantly reduces the diameter of DMVs, causes their aggregation and impairs HCV replication [38]. DMV induction by the distantly related picornaviruses also requires PI4P and hence argues for an evolutionarily conserved mechanism [83]. It is noteworthy that NS5A performs a dual function to manipulate the PI4P metabolism (i) by activating PI4KIIIα and promoting PI4P synthesis at the MW and (ii) by recruiting ARFGAP1 (involved in membrane trafficking) via a tetra-arginine motif in NS5A domain 3 to displace PI4P phosphatase Sac1 and slow down the PI4P turnover rate [84]. Sac1 normally inhibits HCV replication, but NS5A can maintain the PI4P-enriched viral replication microenvironment in favor of HCV replication via its interaction with ARF-GAP1 [84]. PI4P is also synthesized by PI4KIIIβ and is important for HCV replication too [68]. Interestingly, NS5A does not interact with PI4KIIIβ [39]. This also demonstrates the fine-tuned interplay of NS5A with PI4KIIIα in combination with other host proteins involved in PI4P metabolism to maintain a certain microenvironment needed for optimal viral replication and particle assembly in HCV.

The role of NS5A–PI4KIIIα interaction is not limited to induction and modification of the lipid composition of the MW, but extends to the control of HCV pathogenesis. Thus, NS5A cooperates with PI4KIIIα to induce mitochondrial fragmentation, providing cells with higher resistance to mitochondria mediated apoptosis [85] and increasing the probability of developing hepatocellular carcinoma.

## 4. Structure-Function Relationship of NS5A and Role of the Lipid Environment

Despite clear biological evidence demonstrating the importance of phospholipids in HCV replication, there is still a large gap in understanding the underlying structural biological and molecular mechanisms. Functional studies of viral proteins in their lipid environment are particularly difficult. Most studies working on NS5A omit the N-terminal membrane attachment region and the linker to domain 1 to avoid all kinds of technical and methodological problems, in particular purification problems, including insolubility, aggregation, etc. As a consequence, fundamental questions remain on lipid-NS5A interactions and the role of the lipid environment and phospholipids as pivotal regulators in NS5A functional fine-tuning and the biochemistry of full-length NS5A. Although considerable methodological progress has been made over recent years to study membrane proteins and despite their paramount importance in all kinds of biological processes and in biomedical research, most details on their interplay with the lipid phase remain elusive. NS5A still is among the most enigmatic HCV proteins. The 447 residue membrane-anchored protein has a multi-domain architecture and behaves like an integral membrane protein [86]. Domain 1 of NS5A (NS5A-D1) is tethered to intracellular membranes via its N-terminal AH, comprising a membrane-embedded hydrophobic face, the BAAPP motif, that interacts with PIP2 [43], and a polar-charged face exposed to the cytosol that potentially mediates protein–protein interactions [87] (Figure 2A). Working with NS5A is challenging due to its hydrophobic/amphipathic character which necessitates the use of detergents to dissolve the protein out of membranes and keep it soluble. Moreover, NS5A is composed of large unfolded regions (domains 2 and 3, see below) that will not form any crystal contacts for crystallization and/or their structure will not be visible due to flexibility. The flexibility is biologically important and likely required to change protein conformation for different protein–protein interactions. It might also adapt NS5A for different lipidic environments during replication on the ER and the MW and virus production next to lipid droplets.

Each NS5A (sub)domain is composed of different secondary structure elements which require different structural biology methods. Parts of the protein structure are solved by NMR and others by X-ray crystallography, while the full-length structure is still unknown. The N-terminal AH (aa 1-31) is embedded “in-plane” into the membrane. The AH structure was solved by NMR spectroscopy and revealed an amphipathic α-helix (aa 5-25, PDB 1R7E) [87]. The peptide was dissolved in detergent (SDS and dodecylphosphocholine) or in triflouroethanol. Interestingly, next to the helical portion of AH, some flexibility was detected around Val15 and Ser17 [87] and predicted as a kink from residue Val15 to Asp18 [88]. Although Park et al. applied a lipid bilayer membrane platform, only PC and PE were used as common lipid composition for artificial vesicles. The protein structure of NS5A-D1 was solved by several independent labs using X-ray crystallography (PDB 1ZHI, 3FQM, 4CL1) [77,78,79]. Importantly, the three structural models revealed identical D1 monomer conformations but differed in their dimer organization. Every lab used an N-terminally truncated construct of NS5A-D1 that differed in length. The membrane-attachment region was deleted and the structures were solved without a lipidic environment. Tellinghuisen et al. used a fragment with residues 25–215 from genotype 1b Con1, whereas Love et al. used a shorter part including residues 33–202 [77,78]. Unfortunately, the position of residues 25–35 (linker AH to D1) and 199–215 are not resolved in the crystal structure [77]. NS5A-D1 comprises anti-parallel β-sheets containing the zinc-binding site and a short α-helix between the 2 β-strands [77]. Another α-helix at the end of D1 (gt1b, aa 261–266) was identified only by NMR studies [89,90] as this region was not part of the crystallization constructs. The truncated sequences of NS5A-D1 produced monomeric 3D structures that superimposed but showed alternative homodimeric conformations. So far, it remains unclear whether the available crystal structures of N-terminal truncated NS5A-D1 and the related dimer conformations represent true conformational changes and the true conformational space of NS5A respectively or are rather artifacts caused by non-physiological crystallization conditions and the lack of the membrane-attachment region embedded into a (natural) lipid environment. One attempt to shed light into the orientation of the AH towards NS5A-D1 in a lipidic environment using a PE/PC/cholesterol mixture was recently made by Jirasko et al. but again showed the challenge in working with NS5A. Due to high flexibility, AH and its linker to D1 were not visible in the NMR spectra. Nevertheless, the orientation of NS5A-D1 relative to the membrane plane could be determined in this study and revealed a more flat dimer organization than previously observed from crystal structures [91]. Although NS5A-D1 interacts via the highly conserved C-terminal functional interaction site PFIS (Figure 2A) with the lipid kinase PI4KIIIα [76], the physiological importance of PI4P for the function of NS5A in the context of membrane sensing by NS5A-D1 and AH, NS5A-conformational rearrangements and membrane remodeling is still unknown.

Domain 2 and 3 of NS5A are predominantly unfolded. Natively unfolded regions in proteins often serve as a hub for numerous interactions with proteins [92] and NS5A is known to interact with multiple host proteins (reviewed in [93]). Nonetheless, some small structural motifs in D2 and D3 could be identified, e.g., a PW-turn (aa Pro314-Trp316) [94] and transiently formed α-helical segments (upon binding of host interaction partners to D2) [89,90,95,96,97]. In D3, intrinsic α-helical propensities in small regions (around 23–26 aa) and two β-turns (aa region 315–334) were also identified [98,99,100]. The helical region near the N-terminus of D3 (aa JFH-1/Ser360-Gly377 and Con1/Glu365-Gly381) has also an amphipathic character and almost all hydrophobic residues of this helical part are conserved [99]. This helical region might correspond to a molecular recognition element to promote protein–protein interactions [99]. One could also speculate about indirect protein-mediated interactions of D2-3 region with lipids, because i) C-terminal part of NS5A D2 is known to interact with PI3K [101] important for DMVs formation and HCV replication (see above) and ii) connecting D2 and D3 stretch is binding amphiphysin II, which is known to induce membrane curvature [102,103].

Targett-Adams et al. suggested that small molecule inhibitors targeting NS5A (NS5Ai) perturb the function of new replicon complexes rather than acting on preformed complexes, thereby causing a redistribution of NS5A to lipid droplets [104]. NS5Ai are among the most potent classes of DAAs engineered ever with 50% effective concentrations (EC_50_) in replicon assays in the low picomolar range (which corresponds to one molecule of NS5Ai impacting ~ 50,000 molecules of NS5A). It is noteworthy that NS5Ai were not developed by rational drug design, but found via a phenotypic screen based on replication systems [24,105,106]. The bivalent NS5Ais (with two identical pharmacophores) are much more potent than the corresponding monovalent forms. These observations fueled the speculation that bivalent NS5Ai target the dimeric form of NS5A. Based on a model by Nettles et al., NS5Ai bind the NS5A homodimer at the ER-membrane surface with a primary interaction site at residues Tyr93 of both monomers and a lower affinity interaction site near residue Leu31 formed by AH in-plane with the ER membrane. NS5A binding with RNA, NS5B and other proteins induces conformational rearrangements at the membrane interface and AH associated with membrane bending toward replication complex formation (negative-curvature induction) (Figure 2B) that lowers affinity for drug binding. The conformational rearrangements at AH exposes another drug interaction site at a hinge region between Tyr93 and Leu31 of the two different NS5A monomers. NS5Ai binding to this hinge site locks the NS5A dimer in a conformation conductive to lipid droplet formation (positive-curvature induction) (Figure 2B). This mode of action is likely associated with the high activity of NS5Ai by blocking oligomerization [107].

Membrane curvature induction shapes complex membranes to define local subregions and is of central importance for cellular function and membrane trafficking. Generating local membrane curvature is an active process that is mediated and controlled by specialized proteins and general mechanisms, among others by changes in (local) (phospho)lipid composition, reversible interaction with hydrophobic protein regions and nanoscopic scaffolding by oligomerized hydrophilic protein domains [108]. The important role that the lipid phase and phospholipids are likely to play in NS5A is underlined by the observation that resistance mutations against NS5Ai and related fitness-compensatory mutations exclusively occur at or close to the membrane-attachment regions of the protein.

Unraveling the 3D structure of full-length NS5A in its natural lipid environment is a prerequisite for understanding the detailed mechanistic function of this protein and NS5A oligomers formed during HCV replication as well as the exact mode of action of NS5Ai. HCV here is an excellent paradigm for understanding basic principles driving membrane sensing and remodeling in RNA viruses and for understanding the role of lipids in virus biology and pathobiology.

## Figures and Tables

**Table 1:** Phospholipids directly binding to NS proteins of HCV.

HCV Protein	Domain	Phospholipids	Reference(s)
NS4B	AH2	PI4P, PIP2, PIP3, PG ^1^, PS ^2^, CL ^3^	[41,42]
NS5A	AH (BAAPP)	PIP2	[43,44]
NS5B	SBD	SM ^4^	[45]

^1^ Phosphatidylglycerol, ^2^ Phosphatidylserine, ^3^ Cardiolipin, ^4^ Sphingomyelin.

## Abbreviations

aa	amino acid
AH	amphipathic helix
BAAPP	basic amino acid PIP2 pincer
BMRB	Biological Magnetic Resonance Data Bank
CERT	ceramide transfer protein; DFCP1, double FYVE-containing protein 1
DMV	double membrane vesicles; DRM, detergent-resistant membrane
FAPP2	four-phosphate adaptor protein
HCV	hepatitis C virus
IRES	internal ribosome entry site
LD	lipid droplet
LVP	lipo-viro-particle
MMV	multimembrane vesicles
MW	membranous web
NCP	negatively charged phospholipid
NMR	nuclear magnetic resonance
NS	nonstructural
NS5Ai	NS5A inhibitor
ORF	open reading frame
OSBP	oxysterol-binding protein
PDB	Protein Data Bank
PI	phosphatidylinositol
PI3P	phosphatidylinositol 3-phosphate
PI4P	phosphatidylinositol 4-phosphate
PIP2	phosphatidylinositol (4,5)-bisphosphate
PIP3	phosphatidylinositol (3,4,5)-trisphosphate
PI4KIIIα	phosphatidylinositol 4-kinase III alpha
RNA	ribonucleic acid
siRNA	small interfering ribonucleic acid
SM	sphingomyelin
SREBP	sterol regulatory element-binding protein
UTR	untranslated region

## References

[1] Zhang J., Lan Y., Sanyal S. (2017). Modulation of Lipid Droplet Metabolism—A Potential Target for Therapeutic Intervention in Flaviviridae Infections. Front. Microbiol..

[2] Neufeldt C.J., Cortese M., Acosta E.G., Bartenschlager R. (2018). Rewiring Cellular Networks by Members of the Flaviviridae Family. Nat. Rev. Microbiol..

[3] Gosert R., Egger D., Lohmann V., Bartenschlager R., Blum H.E., Bienz K., Moradpour D. (2003). Identification of the Hepatitis C Virus RNA Replication Complex in Huh-7 Cells Harboring Subgenomic Replicons. J. Virol..

[4] Paul D., Hoppe S., Saher G., Krijnse-Locker J., Bartenschlager R. (2013). Morphological and Biochemical Characterization of the Membranous Hepatitis C Virus Replication Compartment. J. Virol..

[5] Romero-Brey I., Berger C., Kallis S., Kolovou A., Paul D., Lohmann V., Bartenschlager R. (2015). NS5A Domain 1 and Polyprotein Cleavage Kinetics Are Critical for Induction of Double-Membrane Vesicles Associated with Hepatitis C Virus Replication. mBio.

[6] Diamond D.L., Syder A.J., Jacobs J.M., Sorensen C.M., Walters K.-A., Proll S.C., McDermott J.E., Gritsenko M.A., Zhang Q., Zhao R. (2010). Temporal Proteome and Lipidome Profiles Reveal Hepatitis C Virus-Associated Reprogramming of Hepatocellular Metabolism and Bioenergetics. PLoS Pathog..

[7] Hofmann S., Krajewski M., Scherer C., Scholz V., Mordhorst V., Truschow P., Schöbel A., Reimer R., Schwudke D., Herker E. (2018). Complex Lipid Metabolic Remodeling Is Required for Efficient Hepatitis C Virus Replication. Biochim. Biophys. Acta Mol. Cell Biol. Lipids.

[8] Bartenschlager R., Lohmann V., Penin F. (2013). The Molecular and Structural Basis of Advanced Antiviral Therapy for Hepatitis C Virus Infection. Nat. Rev. Microbiol..

[9] Moradpour D., Penin F. (2013). Hepatitis C Virus Proteins: From Structure to Function. Curr. Top. Microbiol. Immunol..

[10] Lohmann V., Körner F., Koch J.-O., Herian U., Theilmann L., Bartenschlager R. (1999). Replication of Subgenomic Hepatitis C Virus RNAs in a Hepatoma Cell Line. Science.

[11] Niepmann M., Gerresheim G.K. (2020). Hepatitis C Virus Translation Regulation. Int. J. Mol. Sci..

[12] Dultz G., Shimakami T., Schneider M., Murai K., Yamane D., Marion A., Zeitler T.M., Stross C., Grimm C., Richter R.M. (2020). Extended Interaction Networks with HCV Protease NS3–4A Substrates Explain the Lack of Adaptive Capability against Protease Inhibitors. J. Biol. Chem..

[13] Shimakami T., Welsch C., Yamane D., McGivern D.R., Yi M., Zeuzem S., Lemon S.M. (2011). Protease Inhibitor-Resistant Hepatitis C Virus Mutants with Reduced Fitness from Impaired Production of Infectious Virus. Gastroenterology.

[14] Welsch C., Shimakami T., Hartmann C., Yang Y., Domingues F.S., Lengauer T., Zeuzem S., Lemon S.M. (2012). Peptidomimetic Escape Mechanisms Arise via Genetic Diversity in the Ligand-Binding Site of the Hepatitis C Virus NS3/4A Serine Protease. Gastroenterology.

[15] Doncheva N.T., Domingues F.S., McGivern D.R., Shimakami T., Zeuzem S., Lengauer T., Lange C.M., Albrecht M., Welsch C. (2019). Near-Neighbor Interactions in the NS3–4A Protease of HCV Impact Replicative Fitness of Drug-Resistant Viral Variants. J. Mol. Biol..

[16] Egger D., Wölk B., Gosert R., Bianchi L., Blum H.E., Moradpour D., Bienz K. (2002). Expression of Hepatitis C Virus Proteins Induces Distinct Membrane Alterations Including a Candidate Viral Replication Complex. J. Virol..

[17] Romero-Brey I., Merz A., Chiramel A., Lee J.-Y., Chlanda P., Haselman U., Santarella-Mellwig R., Habermann A., Hoppe S., Kallis S. (2012). Three-Dimensional Architecture and Biogenesis of Membrane Structures Associated with Hepatitis C Virus Replication. PLoS Pathog..

[18] Kandangwa M., Liu Q. (2019). HCV NS5A Hyperphosphorylation Is Involved in Viral Translation Modulation. Biochem. Biophys. Res. Commun..

[19] Shanmugam S., Nichols A.K., Saravanabalaji D., Welsch C., Yi M. (2018). HCV NS5A Dimer Interface Residues Regulate HCV Replication by Controlling Its Self-Interaction, Hyperphosphorylation, Subcellular Localization and Interaction with Cyclophilin, A. PLoS Pathog..

[20] Pawlotsky J.-M., Negro F., Aghemo A., Berenguer M., Dalgard O., Dusheiko G., Marra F., Puoti M., Wedemeyer H. (2018). EASL Recommendations on Treatment of Hepatitis C 2018. J. Hepatol..

[21] Bhattacharjee C., Singh M., Das D., Chaudhuri S., Mukhopadhyay A. (2021). Current Therapeutics against HCV. Virusdisease.

[22] Cotter T.G., Jensen D.M. (2019). Glecaprevir/Pibrentasvir for the Treatment of Chronic Hepatitis C: Design, Development and Place in Therapy. Drug Des. Dev. Ther..

[23] Sarrazin C., Zimmermann T., Berg T., Hinrichsen H., Mauss S., Wedemeyer H., Zeuzem S., Deutsche Gesellschaft für Gastroenterologie, Verdauungs- und Stoffwechselkrankheiten (DGVS), Deutsche Gesellschaft für Pathologie e.V. (DGP) und Bundesverband Deutscher Pathologen (BDP), Deutsche Leberstiftung (2020). Prophylaxe, Diagnostik und Therapie der Hepatitis-C-Virus(HCV)-Infektion. Z. Gastroenterol..

[24] Gao M., Nettles R.E., Belema M., Snyder L.B., Nguyen V.N., Fridell R.A., Serrano-Wu M.H., Langley D.R., Sun J.-H., O’Boyle D.R. (2010). Chemical Genetics Strategy Identifies an HCV NS5A Inhibitor with a Potent Clinical Effect. Nature.

[25] Feld J.J., Foster G.R. (2016). Second Generation Direct-Acting Antivirals—Do We Expect Major Improvements?. J. Hepatol..

[26] Sun J.-H., O’Boyle D.R., Fridell R.A., Langley D.R., Wang C., Roberts S.B., Nower P., Johnson B.M., Moulin F., Nophsker M.J. (2015). Resensitizing Daclatasvir-Resistant Hepatitis C Variants by Allosteric Modulation of NS5A. Nature.

[27] Feng J.Y., Ray A.S. (2021). HCV RdRp, Sofosbuvir and Beyond. Enzymes.

[28] Wolff G., Melia C.E., Snijder E.J., Bárcena M. (2020). Double-Membrane Vesicles as Platforms for Viral Replication. Trends Microbiol..

[29] Ferraris P., Blanchard E., Roingeard P. (2010). Ultrastructural and Biochemical Analyses of Hepatitis C Virus-Associated Host Cell Membranes. J. Gen. Virol..

[30] Ferraris P., Beaumont E., Uzbekov R., Brand D., Gaillard J., Blanchard E., Roingeard P. (2013). Sequential Biogenesis of Host Cell Membrane Rearrangements Induced by Hepatitis C Virus Infection. Cell. Mol. Life Sci. CMLS.

[31] Aizaki H., Lee K.-J., Sung V.M.-H., Ishiko H., Lai M.M.C. (2004). Characterization of the Hepatitis C Virus RNA Replication Complex Associated with Lipid Rafts. Virology.

[32] Quinkert D., Bartenschlager R., Lohmann V. (2005). Quantitative Analysis of the Hepatitis C Virus Replication Complex. J. Virol..

[33] Miyanari Y., Hijikata M., Yamaji M., Hosaka M., Takahashi H., Shimotohno K. (2003). Hepatitis C Virus Non-Structural Proteins in the Probable Membranous Compartment Function in Viral Genome Replication. J. Biol. Chem..

[34] Scutigliani E.M., Kikkert M. (2017). Interaction of the Innate Immune System with Positive-Strand RNA Virus Replication Organelles. Cytokine Growth Factor Rev..

[35] Paul D., Madan V., Bartenschlager R. (2014). Hepatitis C Virus RNA Replication and Assembly: Living on the Fat of the Land. Cell Host Microbe.

[36] Eyre N.S., Fiches G.N., Aloia A.L., Helbig K.J., McCartney E.M., McErlean C.S.P., Li K., Aggarwal A., Turville S.G., Beard M.R. (2014). Dynamic Imaging of the Hepatitis C Virus NS5A Protein during a Productive Infection. J. Virol..

[37] Wang H., Tai A.W. (2017). Continuous de Novo Generation of Spatially Segregated Hepatitis C Virus Replication Organelles Revealed by Pulse-Chase Imaging. J. Hepatol..

[38] Reiss S., Rebhan I., Backes P., Romero-Brey I., Erfle H., Matula P., Kaderali L., Poenisch M., Blankenburg H., Hiet M.-S. (2011). Recruitment and Activation of a Lipid Kinase by Hepatitis C Virus NS5A Is Essential for Integrity of the Membranous Replication Compartment. Cell Host Microbe.

[39] Tai A.W., Salloum S. (2011). The Role of the Phosphatidylinositol 4-Kinase PI4KA in Hepatitis C Virus-Induced Host Membrane Rearrangement. PLoS ONE.

[40] Lyn R.K., Singaravelu R., Kargman S., O’Hara S., Chan H., Oballa R., Huang Z., Jones D.M., Ridsdale A., Russell R.S. (2014). Stearoyl-CoA Desaturase Inhibition Blocks Formation of Hepatitis C Virus-Induced Specialized Membranes. Sci. Rep..

[41] Palomares-Jerez M.F., Nemesio H., Franquelim H.G., Castanho M.A.R.B., Villalaín J. (2013). N-Terminal AH2 Segment of Protein NS4B from Hepatitis C Virus. Binding to and Interaction with Model Biomembranes. Biochim. Biophys. Acta.

[42] Ashworth Briggs E.L., Gomes R.G.B., Elhussein M., Collier W., Findlow I.S., Khalid S., McCormick C.J., Williamson P.T.F. (2015). Interaction between the NS4B Amphipathic Helix, AH2, and Charged Lipid Headgroups Alters Membrane Morphology and AH2 Oligomeric State--Implications for the Hepatitis C Virus Life Cycle. Biochim. Biophys. Acta.

[43] Cho N.-J., Lee C., Pang P.S., Pham E.A., Fram B., Nguyen K., Xiong A., Sklan E.H., Elazar M., Koytak E.S. (2015). Phosphatidylinositol 4,5-Bisphosphate Is an HCV NS5A Ligand and Mediates Replication of the Viral Genome. Gastroenterology.

[44] Kim S.-O., Jackman J.A., Elazar M., Cho S.-J., Glenn J.S., Cho N.-J. (2017). Quantitative Evaluation of Viral Protein Binding to Phosphoinositide Receptors and Pharmacological Inhibition. Anal. Chem..

[45] Sakamoto H., Okamoto K., Aoki M., Kato H., Katsume A., Ohta A., Tsukuda T., Shimma N., Aoki Y., Arisawa M. (2005). Host Sphingolipid Biosynthesis as a Target for Hepatitis C Virus Therapy. Nat. Chem. Biol..

[46] Gouttenoire J., Montserret R., Paul D., Castillo R., Meister S., Bartenschlager R., Penin F., Moradpour D. (2014). Aminoterminal Amphipathic α-Helix AH1 of Hepatitis C Virus Nonstructural Protein 4B Possesses a Dual Role in RNA Replication and Virus Production. PLoS Pathog..

[47] Weng L., Hirata Y., Arai M., Kohara M., Wakita T., Watashi K., Shimotohno K., He Y., Zhong J., Toyoda T. (2010). Sphingomyelin Activates Hepatitis C Virus RNA Polymerase in a Genotype-Specific Manner. J. Virol..

[48] Hirata Y., Ikeda K., Sudoh M., Tokunaga Y., Suzuki A., Weng L., Ohta M., Tobita Y., Okano K., Ozeki K. (2012). Self-Enhancement of Hepatitis C Virus Replication by Promotion of Specific Sphingolipid Biosynthesis. PLoS Pathog..

[49] Forni D., Cagliani R., Pontremoli C., Pozzoli U., Vertemara J., De Gioia L., Clerici M., Sironi M. (2018). Evolutionary Analysis Provides Insight into the Origin and Adaptation of HCV. Front. Microbiol..

[50] Shi S.T., Lee K.-J., Aizaki H., Hwang S.B., Lai M.M.C. (2003). Hepatitis C Virus RNA Replication Occurs on a Detergent-Resistant Membrane That Cofractionates with Caveolin-2. J. Virol..

[51] Sezgin E., Levental I., Mayor S., Eggeling C. (2017). The Mystery of Membrane Organization: Composition, Regulation and Roles of Lipid Rafts. Nat. Rev. Mol. Cell Biol..

[52] Gewaid H., Aoyagi H., Arita M., Watashi K., Suzuki R., Sakai S., Kumagai K., Yamaji T., Fukasawa M., Kato F. (2020). Sphingomyelin Is Essential for the Structure and Function of the Double-Membrane Vesicles in Hepatitis C Virus RNA Replication Factories. J. Virol..

[53] Wang H., Perry J.W., Lauring A.S., Neddermann P., De Francesco R., Tai A.W. (2014). Oxysterol-Binding Protein Is a Phosphatidylinositol 4-Kinase Effector Required for HCV Replication Membrane Integrity and Cholesterol Trafficking. Gastroenterology.

[54] Antonny B., Bigay J., Mesmin B. (2018). The Oxysterol-Binding Protein Cycle: Burning Off PI(4)P to Transport Cholesterol. Annu. Rev. Biochem..

[55] Khan I., Katikaneni D.S., Han Q., Sanchez-Felipe L., Hanada K., Ambrose R.L., Mackenzie J.M., Konan K.V. (2014). Modulation of Hepatitis C Virus Genome Replication by Glycosphingolipids and Four-Phosphate Adaptor Protein 2. J. Virol..

[56] Delang L., Harak C., Benkheil M., Khan H., Leyssen P., Andrews M., Lohmann V., Neyts J. (2018). PI4KIII Inhibitor Enviroxime Impedes the Replication of the Hepatitis C Virus by Inhibiting PI3 Kinases. J. Antimicrob. Chemother..

[57] Twu W.-I., Lee J.-Y., Kim H., Prasad V., Cerikan B., Haselmann U., Tabata K., Bartenschlager R. (2021). Contribution of Autophagy Machinery Factors to HCV and SARS-CoV-2 Replication Organelle Formation. Cell Rep..

[58] Mohl B.-P., Bartlett C., Mankouri J., Harris M. (2016). Early Events in the Generation of Autophagosomes Are Required for the Formation of Membrane Structures Involved in Hepatitis C Virus Genome Replication. J. Gen. Virol..

[59] Bochkov V.N., Oskolkova O.V., Birukov K.G., Levonen A.-L., Binder C.J., Stöckl J. (2010). Generation and Biological Activities of Oxidized Phospholipids. Antioxid. Redox Signal..

[60] Pizzimenti S., Ciamporcero E., Daga M., Pettazzoni P., Arcaro A., Cetrangolo G., Minelli R., Dianzani C., Lepore A., Gentile F. (2013). Interaction of Aldehydes Derived from Lipid Peroxidation and Membrane Proteins. Front. Physiol..

[61] Yamane D., McGivern D.R., Wauthier E., Yi M., Madden V.J., Welsch C., Antes I., Wen Y., Chugh P.E., McGee C.E. (2014). Regulation of the Hepatitis C Virus RNA Replicase by Endogenous Lipid Peroxidation. Nat. Med..

[62] Park C.-Y., Jun H.-J., Wakita T., Cheong J.H., Hwang S.B. (2009). Hepatitis C Virus Nonstructural 4B Protein Modulates Sterol Regulatory Element-Binding Protein Signaling via the AKT Pathway. J. Biol. Chem..

[63] Yang W., Hood B.L., Chadwick S.L., Liu S., Watkins S.C., Luo G., Conrads T.P., Wang T. (2008). Fatty Acid Synthase Is Up-Regulated during Hepatitis C Virus Infection and Regulates Hepatitis C Virus Entry and Production. Hepatology.

[64] Nasheri N., Joyce M., Rouleau Y., Yang P., Yao S., Tyrrell D.L., Pezacki J.P. (2013). Modulation of Fatty Acid Synthase Enzyme Activity and Expression during Hepatitis C Virus Replication. Chem. Biol..

[65] Borawski J., Troke P., Puyang X., Gibaja V., Zhao S., Mickanin C., Leighton-Davies J., Wilson C.J., Myer V., Cornellataracido I. (2009). Class III Phosphatidylinositol 4-Kinase Alpha and Beta Are Novel Host Factor Regulators of Hepatitis C Virus Replication. J. Virol..

[66] Hsu N.-Y., Ilnytska O., Belov G., Santiana M., Chen Y.-H., Takvorian P.M., Pau C., van der Schaar H., Kaushik-Basu N., Balla T. (2010). Viral Reorganization of the Secretory Pathway Generates Distinct Organelles for RNA Replication. Cell.

[67] Berger K.L., Kelly S.M., Jordan T.X., Tartell M.A., Randall G. (2011). Hepatitis C Virus Stimulates the Phosphatidylinositol 4-Kinase III Alpha-Dependent Phosphatidylinositol 4-Phosphate Production That Is Essential for Its Replication. J. Virol..

[68] Zhang L., Hong Z., Lin W., Shao R.-X., Goto K., Hsu V.W., Chung R.T. (2012). ARF1 and GBF1 Generate a PI4P-Enriched Environment Supportive of Hepatitis C Virus Replication. PLoS ONE.

[69] Furse S., Brooks N.J., Seddon A.M., Woscholski R., Templer R.H., Tate E.W., Gaffney P.R.J., Ces O. (2012). Lipid Membrane Curvature Induced by Distearoyl Phosphatidylinositol 4-Phosphate. Soft Matter.

[70] Godi A., Pertile P., Meyers R., Marra P., Di Tullio G., Iurisci C., Luini A., Corda D., De Matteis M.A. (1999). ARF Mediates Recruitment of PtdIns-4-OH Kinase-Beta and Stimulates Synthesis of PtdIns(4,5)P2 on the Golgi Complex. Nat. Cell Biol..

[71] Bishé B., Syed G., Siddiqui A. (2012). Phosphoinositides in the Hepatitis C Virus Life Cycle. Viruses.

[72] Blumental-Perry A., Haney C.J., Weixel K.M., Watkins S.C., Weisz O.A., Aridor M. (2006). Phosphatidylinositol 4-Phosphate Formation at ER Exit Sites Regulates ER Export. Dev. Cell.

[73] Bianco A., Reghellin V., Donnici L., Fenu S., Alvarez R., Baruffa C., Peri F., Pagani M., Abrignani S., Neddermann P. (2012). Metabolism of Phosphatidylinositol 4-Kinase IIIα-Dependent PI4P Is Subverted by HCV and Is Targeted by a 4-Anilino Quinazoline with Antiviral Activity. PLoS Pathog..

[74] Lim Y.-S., Hwang S.B. (2011). Hepatitis C Virus NS5A Protein Interacts with Phosphatidylinositol 4-Kinase Type IIIalpha and Regulates Viral Propagation. J. Biol. Chem..

[75] Ahn J., Chung K.-S., Kim D.-U., Won M., Kim L., Kim K.-S., Nam M., Choi S.-J., Kim H.-C., Yoon M. (2004). Systematic Identification of Hepatocellular Proteins Interacting with NS5A of the Hepatitis C Virus. J. Biochem. Mol. Biol..

[76] Reiss S., Harak C., Romero-Brey I., Radujkovic D., Klein R., Ruggieri A., Rebhan I., Bartenschlager R., Lohmann V. (2013). The Lipid Kinase Phosphatidylinositol-4 Kinase III Alpha Regulates the Phosphorylation Status of Hepatitis C Virus NS5A. PLoS Pathog..

[77] Tellinghuisen T.L., Marcotrigiano J., Rice C.M. (2005). Structure of the Zinc-Binding Domain of an Essential Component of the Hepatitis C Virus Replicase. Nature.

[78] Love R.A., Brodsky O., Hickey M.J., Wells P.A., Cronin C.N. (2009). Crystal Structure of a Novel Dimeric Form of NS5A Domain I Protein from Hepatitis C Virus. J. Virol..

[79] Lambert S.M., Langley D.R., Garnett J.A., Angell R., Hedgethorne K., Meanwell N.A., Matthews S.J. (2014). The Crystal Structure of NS5A Domain 1 from Genotype 1a Reveals New Clues to the Mechanism of Action for Dimeric HCV Inhibitors. Protein Sci. Publ. Protein Soc..

[80] Harak C., Radujkovic D., Taveneau C., Reiss S., Klein R., Bressanelli S., Lohmann V. (2014). Mapping of Functional Domains of the Lipid Kinase Phosphatidylinositol 4-Kinase Type III Alpha Involved in Enzymatic Activity and Hepatitis C Virus Replication. J. Virol..

[81] Wong M.-T., Chen S.S. (2017). Hepatitis C Virus Subverts Human Choline Kinase-α To Bridge Phosphatidylinositol-4-Kinase IIIα (PI4KIIIα) and NS5A and Upregulates PI4KIIIα Activation, Thereby Promoting the Translocation of the Ternary Complex to the Endoplasmic Reticulum for Viral Replication. J. Virol..

[82] Reghellin V., Donnici L., Fenu S., Berno V., Calabrese V., Pagani M., Abrignani S., Peri F., De Francesco R., Neddermann P. (2014). NS5A Inhibitors Impair NS5A-Phosphatidylinositol 4-Kinase IIIα Complex Formation and Cause a Decrease of Phosphatidylinositol 4-Phosphate and Cholesterol Levels in Hepatitis C Virus-Associated Membranes. Antimicrob. Agents Chemother..

[83] Altan-Bonnet N., Balla T. (2012). Phosphatidylinositol 4-Kinases: Hostages Harnessed to Build Panviral Replication Platforms. Trends Biochem. Sci..

[84] Li H., Yang X., Yang G., Hong Z., Zhou L., Yin P., Xiao Y., Chen L., Chung R.T., Zhang L. (2014). Hepatitis C Virus NS5A Hijacks ARFGAP1 to Maintain a Phosphatidylinositol 4-Phosphate-Enriched Microenvironment. J. Virol..

[85] Siu G.K.Y., Zhou F., Yu M.K., Zhang L., Wang T., Liang Y., Chen Y., Chan H.C., Yu S. (2016). Hepatitis C Virus NS5A Protein Cooperates with Phosphatidylinositol 4-Kinase IIIα to Induce Mitochondrial Fragmentation. Sci. Rep..

[86] Brass V., Bieck E., Montserret R., Wölk B., Hellings J.A., Blum H.E., Penin F., Moradpour D. (2002). An Amino-Terminal Amphipathic Alpha-Helix Mediates Membrane Association of the Hepatitis C Virus Nonstructural Protein 5A. J. Biol. Chem..

[87] Penin F., Brass V., Appel N., Ramboarina S., Montserret R., Ficheux D., Blum H.E., Bartenschlager R., Moradpour D. (2004). Structure and Function of the Membrane Anchor Domain of Hepatitis C Virus Nonstructural Protein 5A. J. Biol. Chem..

[88] Park S., Jackman J.A., Cho N.-J. (2019). Comparing the Membrane-Interaction Profiles of Two Antiviral Peptides: Insights into Structure–Function Relationship. Langmuir.

[89] Feuerstein S., Solyom Z., Aladağ A., Hoffmann S., Willbold D., Brutscher B. (2011). 1H, 13C, and 15N Resonance Assignment of a 179 Residue Fragment of Hepatitis C Virus Non-Structural Protein 5A. Biomol. NMR Assign..

[90] Feuerstein S., Solyom Z., Aladag A., Favier A., Schwarten M., Hoffmann S., Willbold D., Brutscher B. (2012). Transient Structure and SH3 Interaction Sites in an Intrinsically Disordered Fragment of the Hepatitis C Virus Protein NS5A. J. Mol. Biol..

[91] Jirasko V., Lends A., Lakomek N.-A., Fogeron M.-L., Weber M., Malär A., Penzel S., Bartenschlager R., Meier B.H., Böckmann A. (2021). Dimer Organization of Membrane-Associated NS5A of Hepatitis C Virus as Determined by Highly Sensitive 1H-Detected Solid-State NMR. Angew. Chem. Int. Ed. Engl..

[92] Uversky V.N., Oldfield C.J., Dunker A.K. (2008). Intrinsically Disordered Proteins in Human Diseases: Introducing the D2 Concept. Annu. Rev. Biophys..

[93] Cordek D.G., Bechtel J.T., Maynard A.T., Kazmierski W.M., Cameron C.E. (2011). Targeting the ns5a protein of hcv: An emerging option. Drugs Future.

[94] Dujardin M., Madan V., Montserret R., Ahuja P., Huvent I., Launay H., Leroy A., Bartenschlager R., Penin F., Lippens G. (2015). A Proline-Tryptophan Turn in the Intrinsically Disordered Domain 2 of NS5A Protein Is Essential for Hepatitis C Virus RNA Replication. J. Biol. Chem..

[95] Liang Y., Ye H., Kang C.B., Yoon H.S. (2007). Domain 2 of Nonstructural Protein 5A (NS5A) of Hepatitis C Virus Is Natively Unfolded. Biochemistry.

[96] Hanoulle X., Badillo A., Verdegem D., Penin F., Lippens G. (2010). The Domain 2 of the HCV NS5A Protein Is Intrinsically Unstructured. Protein Pept. Lett..

[97] Badillo A., Receveur-Brechot V., Sarrazin S., Cantrelle F.-X., Delolme F., Fogeron M.-L., Molle J., Montserret R., Bockmann A., Bartenschlager R. (2017). Overall Structural Model of NS5A Protein from Hepatitis C Virus and Modulation by Mutations Confering Resistance of Virus Replication to Cyclosporin A. Biochemistry.

[98] Hanoulle X., Verdegem D., Badillo A., Wieruszeski J.-M., Penin F., Lippens G. (2009). Domain 3 of Non-Structural Protein 5A from Hepatitis C Virus Is Natively Unfolded. Biochem. Biophys. Res. Commun..

[99] Verdegem D., Badillo A., Wieruszeski J.-M., Landrieu I., Leroy A., Bartenschlager R., Penin F., Lippens G., Hanoulle X. (2011). Domain 3 of NS5A Protein from the Hepatitis C Virus Has Intrinsic Alpha-Helical Propensity and Is a Substrate of Cyclophilin A. J. Biol. Chem..

[100] Sólyom Z., Ma P., Schwarten M., Bosco M., Polidori A., Durand G., Willbold D., Brutscher B. (2015). The Disordered Region of the HCV Protein NS5A: Conformational Dynamics, SH3 Binding, and Phosphorylation. Biophys. J..

[101] Street A., Macdonald A., Crowder K., Harris M. (2004). The Hepatitis C Virus NS5A Protein Activates a Phosphoinositide 3-Kinase-Dependent Survival Signaling Cascade. J. Biol. Chem..

[102] Zech B., Kurtenbach A., Krieger N., Strand D., Blencke S., Morbitzer M., Salassidis K., Cotten M., Wissing J., Obert S. (2003). Identification and Characterization of Amphiphysin II as a Novel Cellular Interaction Partner of the Hepatitis C Virus NS5A Protein. J. Gen. Virol..

[103] Masumi A., Aizaki H., Suzuki T., DuHadaway J.B., Prendergast G.C., Komuro K., Fukazawa H. (2005). Reduction of Hepatitis C Virus NS5A Phosphorylation through Its Interaction with Amphiphysin II. Biochem. Biophys. Res. Commun..

[104] Targett-Adams P., Graham E.J.S., Middleton J., Palmer A., Shaw S.M., Lavender H., Brain P., Tran T.D., Jones L.H., Wakenhut F. (2011). Small Molecules Targeting Hepatitis C Virus-Encoded NS5A Cause Subcellular Redistribution of Their Target: Insights into Compound Modes of Action. J. Virol..

[105] Conte I., Giuliano C., Ercolani C., Narjes F., Koch U., Rowley M., Altamura S., De Francesco R., Neddermann P., Migliaccio G. (2009). Synthesis and SAR of Piperazinyl-N-Phenylbenzamides as Inhibitors of Hepatitis C Virus RNA Replication in Cell Culture. Bioorgan. Med. Chem. Lett..

[106] Lemm J.A., O’Boyle D., Liu M., Nower P.T., Colonno R., Deshpande M.S., Snyder L.B., Martin S.W., St Laurent D.R., Serrano-Wu M.H. (2010). Identification of Hepatitis C Virus NS5A Inhibitors. J. Virol..

[107] Nettles J.H., Stanton R.A., Broyde J., Amblard F., Zhang H., Zhou L., Shi J., McBrayer T.R., Whitaker T., Coats S.J. (2014). Asymmetric Binding to NS5A by Daclatasvir (BMS-790052) and Analogs Suggests Two Novel Modes of HCV Inhibition. J. Med. Chem..

[108] McMahon H.T., Boucrot E. (2015). Membrane Curvature at a Glance. J. Cell Sci..

